# Isolation of Highly Pathogenic Avian Influenza A(H5N1) Virus from Fetal Bovine Serum, United States, 2025

**DOI:** 10.3201/eid3208.260077

**Published:** 2026-08

**Authors:** Ana R. Rebelo, Erica Butler, Elizabeth Aguilera Nunez, Brittany Cronk, Jennifer Powers, Melissa Laverack, Chen Feng, Mohammed Nooruzzaman, Mia K. Torchetti, Leonardo C. Caserta, Diego G. Diel

**Affiliations:** Cornell University College of Veterinary Medicine, Ithaca, New York, USA (A.R. Rebelo, E. Butler, E. Aguilera Nunez, B. Cronk, J. Powers, M. Laverack, C. Feng, M. Nooruzzaman, L.C. Caserta, D.G. Diel); US Department of Agriculture Animal and Plant Health Inspection Service, Ames, Iowa, USA (M.K. Torchetti)

**Keywords:** influenza, viruses, highly pathogenic avian influenza, H5N1, zoonoses, United States

## Abstract

In February 2025, we detected highly pathogenic avian influenza virus A(H5N1) clade 2.3.4.4b virus in a fetal bovine serum lot during routine adventitious agent testing. Sequencing confirmed H5N1 genotype B3.13 virus. We found low viral loads in additional samples from the same lot. Heating at 56°C for 30 minutes completely inactivated the virus.

Highly pathogenic avian influenza (HPAI) A(H5N1) clade 2.3.4.4b genotype B3.13 virus infections were reported in dairy cows in Texas, USA, for the first time in March 2024 (*1*–*3*); rapid virus spread followed. As of December 18, 2025, more than 1,084 herds in 19 US states have been affected; most of the herds are in California (*4*). We report detection of HPAI H5N1 clade 2.3.4.4b genotype B3.13 virus in a fetal bovine serum (FBS) lot during routine testing.

## The Study

In February 2025, a lot of FBS was received by the Virology Laboratory at the Cornell Animal Health Diagnostic Center (AHDC; Ithaca, NY, USA) for routine extraneous agents testing, per requirements of Title 9 of the Code of Federal Regulations (9CFR) for animal origin biologic products (§113.53, §113.46, §113.47). The tested FBS consisted of a pool of samples collected in 5 states (Pennsylvania, Kansas, Nebraska, South Carolina, and California). We tested the sample as prescribed in 9CFR, by seeding Vero cells (ATCC CCL-81) and primary fetal bovine kidney (FBK) cells, developed by the AHDC Virology Laboratory, in T75 tissue culture flasks in Eagle minimum essential medium supplemented with 2% penicillin/streptomycin and 15% (final concentration) of the test FBS sample. We maintained cells for 7 days and monitored for cytopathology. FBK cells did not exhibit viral cytopathic effect (CPE) but were positive for noncytopathic bovine viral diarrhea virus through virus-specific immunofluorescence assay (IFA) staining on day 7. We observed CPE in Vero cells on day 7 post-inoculation. Subsequent IFA testing of the Vero cells was negative for the bovine viruses listed in 9CFR (i.e., bovine viral diarrhea virus, bovine parvovirus, bluetongue virus, reovirus, bovine adenovirus, bovine respiratory syncytial virus, and rabies virus). We tested FBK and Vero cells cultured in the presence of 15% control gamma-irradiated FBS in accordance with 9CFR regulations; the cells remained negative for CPE and IFA virus.

**Figure 1:**
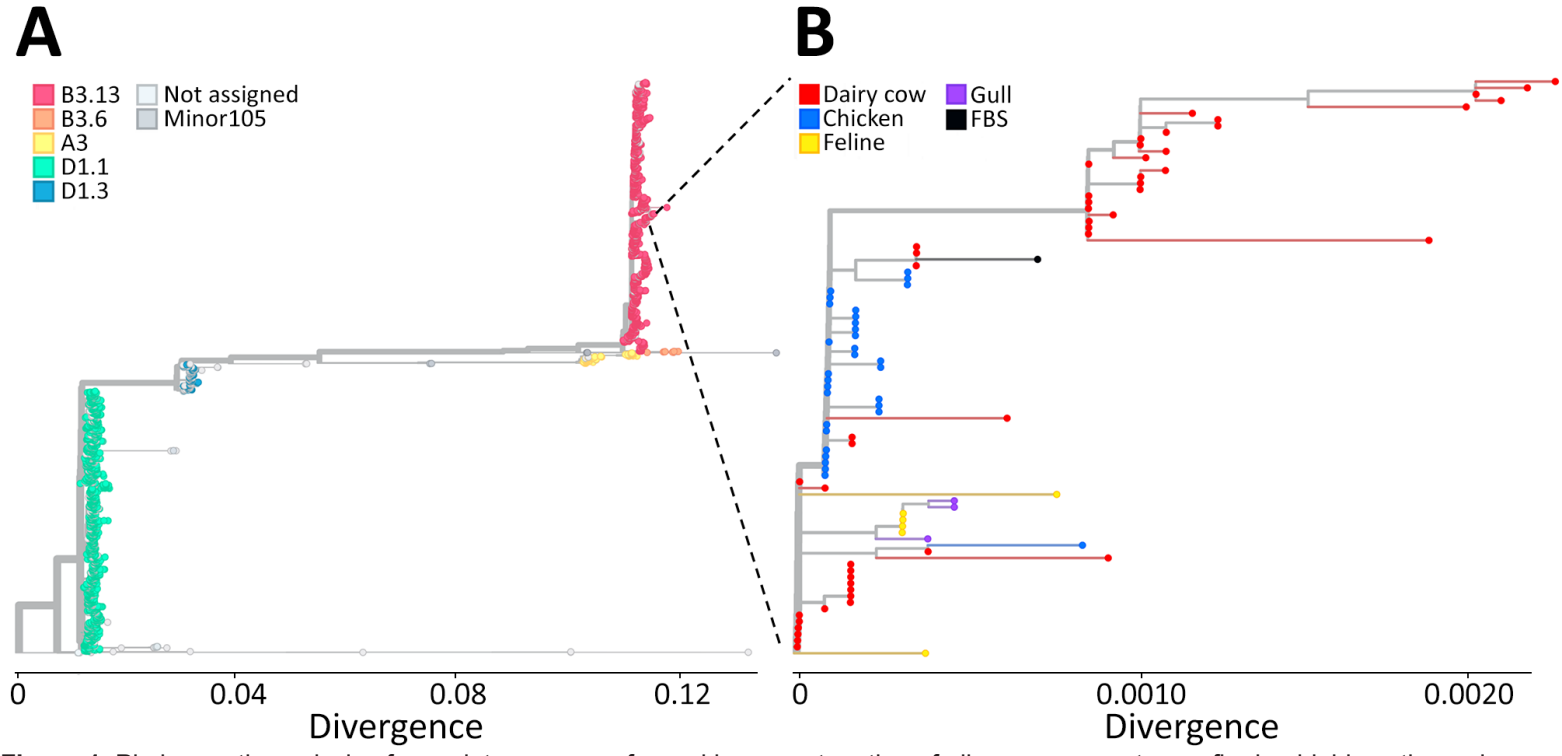
Phylogenetic analysis of complete genomes formed by concatenation of all gene segments, confirming highly pathogenic avian influenza A (H5N1) virus genotype B3.13 isolated from fetal bovine serum (FBS), United States, 2025. A) Tree showing broader phylogeny of H5N1 virus genotypes. B) Closer examination of the virus from FBS sample and closely related virus sequences. Dashed lines indicate the branch in the broader phylogenetic tree where the FBS virus sequence is located. Dataset consisted of 5,094 highly pathogenic avian influenza A(H5N1) virus genomes from samples collected during August 2024–January 2025 in North America, downloaded from the GISAID EpiFlu database (*5*). Maximum-likelihood phylogenetic inferred with an edge-linked partition model and 1,000 bootstrap replicates using IQ-TREE (*6*).

We performed viral metagenomic sequencing of the supernatant obtained from Vero cells showing CPE after conducting sequence-independent, single-primer amplification of nucleic acids and library preparation using the ligation sequencing gDNA kit (SQK-LSK109) and Native Barcoding Kit 96 (Oxford Nanopore Technologies, https://www.nanoporetech.com). We conducted sequencing on a MinION flow cell using the GridION platform (both Oxford Nanopore Technologies). We classified a total of 13,599 reads as avian influenza A virus subtype H5N1. Bioinformatic analysis identified the virus as H5N1 clade 2.3.4.4b genotype B3.13 (GISAID accession nos. EPI4889248–55). Phylogenetic analysis of concatenated gene segment sequences indicated that the FBS H5N1 isolate sequence clustered within the B3.13 genotype and was closely related to dairy cow samples from California collected during December 2024 (Figure 1). We confirmed the presence of avian influenza A virus in this isolate by using real-time reverse transcription PCR (rRT-PCR). Because of the detection of H5N1 virus in this sample, we discontinued adventitious virus testing on the original sample.

**Figure 2:**
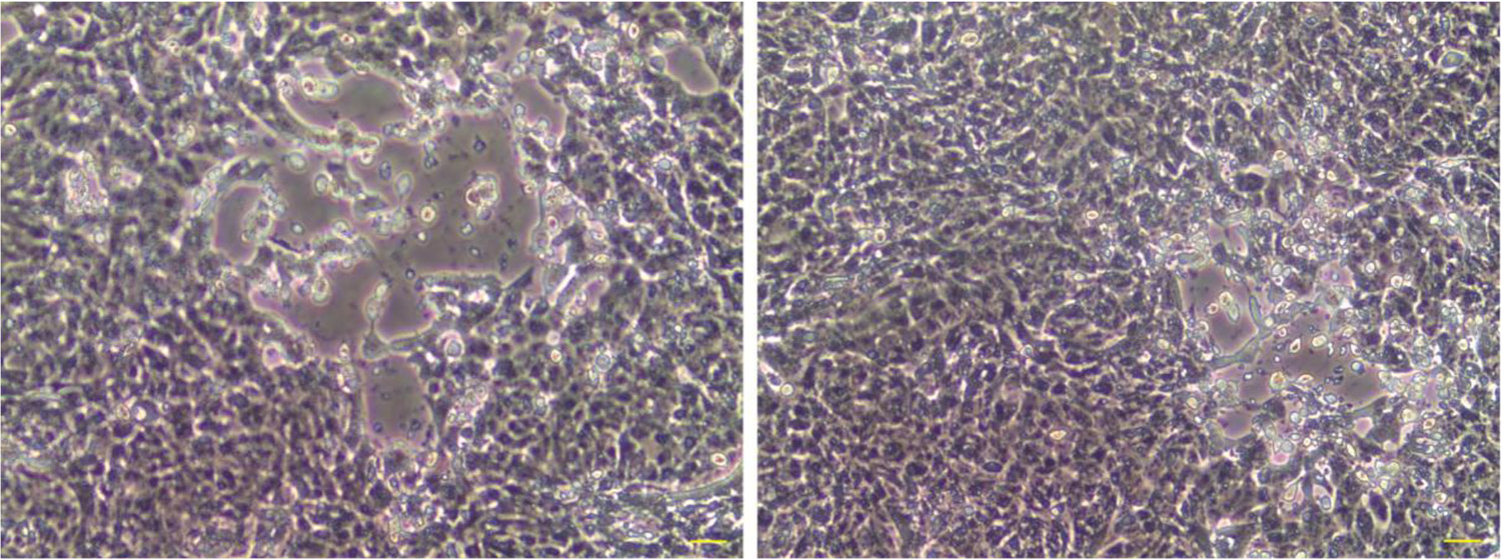
Cytopathic effects of highly pathogenic avian influenza A (H5N1) virus in Vero cells isolated from fetal bovine serum, United States, 2025. Vero cells inoculated with non–heat-inactivated fetal bovine serum sample (046093-25-2) show cell rounding and vacuolation on day 7 of incubation. Images represent different fields of view of the same cell culture on the same day (original magnification × 20).

We performed additional testing, including rRT-PCR and virus isolation in cell culture and embryonated chicken eggs (ECE), on the same lot of FBS by using 8 newly submitted 100-mL samples of non–heat-inactivated and 5 100-mL samples of heat-inactivated (56°C for 30 min) samples to investigate and confirm the presence of HPAI virus subtype H5N1 (Table). We tested all 13 FBS samples by using the National Animal Health Laboratory Network avian influenza A virus matrix rRT-PCR protocol, resulting in viral RNA detection from 1 non–heat-inactivated sample (046093-25-4) (cycle threshold [Ct] 37.7) and 1 heat-inactivated sample (046093-25-9) (Ct 39.1). For virus isolation in cell culture, we again followed 9CFR prescribed testing and Vero and bovine uterine epithelial cells (Cal-1, known to be susceptible to H5N1 virus) seeded in T75 flasks. We cultured the cells in the presence of 15% FBS and maintained them for 7 days before the next subculture, for a total of 3 subcultures (total of 21 days). We monitored cells daily for CPE. One of the non–heat-inactivated FBS samples (046093-25-2) showed CPE in Vero cells (Figure 2), and we confirmed influenza A virus isolation by using a matrix-specific rRT-PCR (Table).

**Table:** Summary results of further testing of fetal bovine serum samples for highly pathogenic avian influenza A(H5N1) virus, United States, 2025*

Sample no.	Treatment	Cell culture virus isolation†	Embryonated egg virus isolation‡	rRT-PCR
046093-25-1	Not HI	ND	ND	ND
046093-25-2	Not HI	Detected, Ct 9.50	ND	ND
046093-25-3	Not HI	ND	ND	ND
046093-25-4	Not HI	ND	ND	Detected, Ct 37.78
046093-25-5	Not HI	ND	ND	ND
046093-25-6	Not HI	ND	Detected, Ct 4.73 (UC), Ct 31 (10:1 dilution)	ND
046093-25-7	Not HI	ND	Detected, Ct 31 (10:1 dilution)	ND
046093-25-8	Not HI	ND	ND	ND
046093-25-9	HI	ND	ND	Detected, Ct 39.10
046093-25-10	HI	ND	ND	ND
046093-25-11	HI	ND	ND	ND
046093-25-12	HI	ND	ND	ND
046093-25-13	HI	ND	ND	ND

*Thirteen FBS samples were received (8 bottles of non–HI FBS and 5 bottles of HI FBS). Samples were tested by using virus isolation in cell culture (Vero and Cal-1 cell lines), embryonated eggs, and avian influenza virus rRt-PCR. In embryonated eggs, FBS was inoculated undiluted, diluted 10:1, diluted 10:2, and UC. Ct, cycle threshold; ND, not detected; HI, heat inactivated; rRT-PCR, real-time reverse transcription PCR; UC, ultracentrifuged. †H5N1 considered detected if cytopathic effect observed and cell culture supernatant positive by rRT-PCR. ‡H5N1 considered detected if allantoic fluid positive by rRT-PCR and hemagglutination assay.

To increase the sensitivity of the virus isolation, we also tested FBS samples by using ECE virus isolation. We subjected 15 mL of the original samples to ultracentrifugation (32,000 rpm for 1 h 30 min) in a sucrose cushion (10 × NTC buffer: 1 M NaCl, 0.2 M Tris-HCL pH 7.4, 50 mM CaCl2; sucrose 30% [mass/volume]), then resuspended the pellet in 1 mL of 1× phosphate-buffered saline. We inoculated 10-day-old ECEs with 0.3 mL of undiluted and diluted (10:1 and 10:2, phosphate-buffered saline) original samples and 0.1 mL ultracentrifuged FBS samples per egg (3 eggs per condition). We monitored embryo viability daily for 5 days. After 5 days, we collected the allantoic fluid and tested it for avian influenza A virus by using rRT-PCR and a hemagglutination assay and then re-inoculated them into ECEs for a second passage. We isolated virus from 2 non–heat-inactivated, ultracentrifuged FBS samples. Sample 046093-25-6 was positive at a 10^−1^ dilution, so we ultracentrifuged it. We confirmed influenza isolation by matrix rRT-PCR and hemagglutination assay. One sample (046093-25-7) was negative by hemagglutination assay at a 10^−1^ dilution after the first passage in ECE, but the allantoic fluid tested positive by matrix rRT-PCR (Ct 31).

Attempts to confirm virus isolation at the US Department of Agriculture Animal and Plant Health Inspection Service’s National Veterinary Services Laboratories (Ames, IA, USA) were not successful. The low frequency of HPAI H5N1 virus detection in the tested FBS lot (even when tested using rRT-PCR) and the lack of detection of the virus suggest low viral loads (probably <1 infectious particle/mL) in this pooled FBS lot.

Given our findings and a suspicion of viremia in infected lactating dairy cows (*2*,*7*,*8*), we performed further testing of individual FBS samples collected at slaughterhouses in California (n = 384) and Texas (n = 384). Among those samples, 1 tested positive by avian influenza A virus matrix rRT-PCR (Ct 35). This finding in fetal serum supports the hypothesis that sporadic HPAI viremia in dairy cattle enables the virus to reach different tissues (*7*,*8*), including, in rare occasions, the fetus. Reports from dairy farms suggest that heifers infected with H5N1 have experienced reproductive complications, including abortion, after HPAI H5N1 virus infection (*9*).

**Figure 3:**
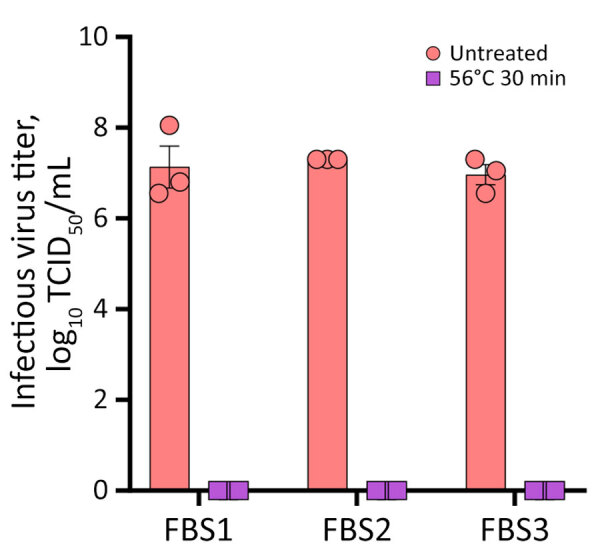
Heat inactivation of highly pathogenic avian influenza A virus subtype H5N1 in FBS, United States, 2025. FBS samples (1-mL aliquots) were spiked (10^7^ TCID_50_/mL) with bovine highly pathogenic avian influenza A (H5N1) virus TX2/24 strain (clade 2.3.4.4b, genotype B3.13) and heat treated at 56°C for 30 minutes by using a water bath. Virus titers determined by endpoint dilutions in Cal-1 cells and expressed as TCID_50_/mL. Data represents observations (dots) from 3 independent experiments overlayed with the mean (horizontal line) +SEM (whiskers). FBS, fetal bovine serum; TCID_50_, 50% tissue culture infectious dose.

Next, we evaluated the efficiency of heat inactivation of FBS for HPAI virus. Thermal treatment of the serum (1-mL aliquots spiked with ≈7 log_10_ 50% tissue culture infectious dose/mL) at 56°C for 30 minutes inactivated the virus (Figure 3).

## Conclusions

FBS is the most frequently used growth supplement for cell culture media. Although the finding of a zoonotic virus in FBS raises biosafety concerns for diagnostic testing of bovine serum and other laboratory procedures that use FBS, such as cell culture, heat treatment of FBS seems to be efficient in inactivating the virus and could be used as a potential safety precaution.
